# Xenotransplantation: Injustice, Harm, and Alternatives for Addressing the Organ Crisis

**DOI:** 10.1002/hast.5021

**Published:** 2025-10-15

**Authors:** Jasmine Gunkel, Franklin G. Miller

**Keywords:** xenotransplantation, animal welfare, health justice, organ shortage, public health interventions, bioethics, research ethics

## Abstract

*Xenotransplantation is increasingly touted as the solution to the organ crisis. Some bioethicists, however, have raised concerns about xenotransplantation's implications for health justice and animal welfare. We develop and sharpen these worries, and we explore how they might be mitigated. We compare xenotransplantation with several alternatives for addressing the organ crisis, including directing more money toward public health interventions, and argue that these alternatives are ethically preferable. In light of this, we argue that xenotransplantation is not a justifiable method of addressing the organ crisis. Societal funds and attention ought instead be directed toward more efficient and ethically superior solutions*.

## Article

Every year, six thousand people in the United States die awaiting organ transplant. Over a hundred thousand more sit on transplant waiting lists.^1^ Time waiting for an organ is often frightening, painful, and disruptive to one's life. For kidneys, the most needed organ, people wait four years on average.^2^ During this wait, patients might spend five hours three times a week at a dialysis center, commonly enduring cramping and itchiness and at risk of many more serious side effects.^3^ Nearly 20 percent of patients waiting for kidneys are removed from the waiting list, commonly because they are deemed too sick for major surgery.^4^ And many people struggle to get on waiting lists at all. While harder to estimate, worldwide need for organs is much greater. The gap between the need for organs and their availability is appropriately known as the “organ crisis.” Xenotransplantation, the practice of transplanting organs across species lines, is increasingly often touted as the best way to close this gap.

Xenotransplantation has been in the public consciousness since at least the 1980s, when Baby Fae lived three weeks with a transplanted baboon heart.^5^ But in recent years, headlines more frequently buzz with excitement about xenotransplantation milestones using organs of genetically modified pigs. There was news of the first transplant of a pig's heart into a human patient, and headlines announced that the first patient to receive a pig kidney went home from the hospital.^6^ Though the first four recipients of pig organs all died within two months of transplant,^7^ xenotransplantation is no longer a science‐fiction ethical issue. For the fifth recipient, the pig kidney lasted over four months before her body rejected it and the organ had to be removed.^8^ And in February 2025, the U.S. Food and Drug Administration (FDA) announced approval of the first two xenotransplantation studies: a larger one set to begin later this year and one that gave a sixth patient a kidney in January 2025.^9^

Many physicians and bioethicists have urged moving forward with clinical trials of xenotransplantation.^10^ Although there has been substantial worry about the potential for zoonotic diseases to jump to humans with such procedures, many scientists and medical professionals seem confident that these risks can be adequately mitigated.^11^ Some commentators have criticized xenotransplantation on animal rights and welfare grounds.^12^ Others have raised alarm bells about the potential for xenotransplantation to worsen health care inequities.^13^ In this paper, we systematically analyze the latter two sets of concerns.

We highlight morally significant ways in which xenotransplantation, particularly if clinically adopted, differs from common harmful uses of animals in research. We argue that the size of the exploited class of genetically modified pigs and relative permanence of their exploitation make the clinical adoption of xenotransplantation particularly ethically fraught.

Second, we consider justice among humans. It's easy to imagine that xenotransplantation, because it increases the supply of organs, will inevitably lead to a more just health system. Many commentators have emphasized its potential to catch those falling through the allotransplantation cracks. However, there are potential pitfalls, both for xenotransplantation research and clinical adoption. Xenotransplantation has the potential to widen existing health care disparities, including by making some individuals not just comparatively but also absolutely worse off. We explore how the potential for such injustices might be mitigated.

Moreover, given the opportunity costs of investing so much money into xenotransplantation, an adequate ethical evaluation must assess how it compares with alternative approaches to address the organ crisis. We argue that low‐tech solutions, like investing in public health and improving organ donation, would be better able to address the organ crisis and promote health. While less attention‐grabbing, these low‐cost solutions are more efficient and just, and they don't rely on harmful exploitation. Finally, we explore technological alternatives, such as growing organs in labs, that, while likely farther away, are ethically superior. Societal funds and attention ought to be directed away from xenotransplantation and toward more ethically justifiable alternatives.

## Caveats

In this paper, we'll imagine that xenotransplantation's risks of introducing zoonoses to human communities can be sufficiently minimized. This is not to dismiss those risks. As Covid‐19 made only too clear, the emergence of a novel disease can be devastating. If the risk of zoonoses cannot be adequately minimized, we see this alone as a decisive reason to halt xenotransplantation research, making our arguments superfluous.

We also assume for the sake of argument that some harmful use of nonhuman animals in biomedical research and clinical practice can be permissible. While one of us (Jasmine Gunkel) leans in the abolitionist direction, if harmful exploitation of animals in research and practice is never justifiable, then there is nothing interesting to say in evaluating xenotransplantation from an animal ethics perspective. Furthermore, we hope to convince a broad audience, including those who believe that some harmful uses of animals are justified, that there are serious ethical problems with pursuing xenotransplantation as the solution to the organ shortage.

## The Organ Crisis and the Benefits of Xenotransplantation

Though access to a functioning organ most obviously benefits those who would die without access, the shortening of wait times is beneficial for everyone on the list. Being on dialysis is logistically complex and physically taxing. Experiencing heart failure restricts your movement and everyday activities. Spending years waiting for an organ, hoping it'll be your turn before you're too sick, takes a tremendous mental toll. Waiting lists for kidneys, hearts, and some other organs could be eliminated in the United States if pigs were raised for organs in large numbers and xenotransplantation became routine.

Although the number of people who die worldwide because they can't receive an organ is higher and harder to estimate, it's plain that many of those deaths would not be averted by xenotransplantation. Countries that perform the fewest number of transplants tend to be the poorest, with fewer health care resources and lower health care spending.^14^ If people are dying from organ failure due to shortages of transplant surgeons, unavailability of immunosuppressants and follow‐up care, and a health care system that is struggling to address even relatively inexpensive medical needs, a supply of pig organs is not a real remedy. Any serious look at the potential benefits of xenotransplantation has to recognize that limited organ supply is only one of the factors that must be addressed to prevent death due to organ failure.

## Opportunity Costs and Priority Setting

While xenotransplantation could save tens of thousands of lives every year and improve the lives of hundreds of thousands of people, like most expensive health care interventions, it would offer little benefit to those outside wealthier countries. Even in well‐resourced settings, transplantation is enormously expensive. The estimated cost for a kidney transplant nears half a million U.S. dollars, while a heart transplant nears 1.7 million.^15^ Countries do not have unlimited funds for health care and health research—a problem those working in health research priority setting have grappled with for decades.^16^ Biotech companies have already poured hundreds of millions into xenotransplantation, and this spending is projected to increase significantly.^17^ There are opportunity costs to researching and adopting expensive interventions rather than other potential technologies that are not brought to fruition and public health measures that don't receive support. A society must think not only about the absolute benefits of an intervention but also about the benefits per dollar spent. An intervention's efficacy, along with its other morally significant features, must be compared with the features of other potential interventions.

In what follows, we engage in this kind of comparative analysis for xenotransplantation. We will not compare addressing the organ crisis to addressing other unmet health care needs. Rather, we will focus exclusively on how xenotransplantation compares to other methods of addressing the organ crisis. If xenotransplantation is an inferior method, as we contend, then the better methods of addressing the organ crisis are the ones that ought to be weighed in larger priority‐setting debates. We begin by addressing some major ethical shortfalls of xenotransplantation and then move to comparing it with alternative methods of addressing the organ crisis.

## Xenotransplantation and Pigs

Given the size of their organs and their similarity to our own, as well as pigs’ quick developmental time, pigs are the animal of choice for xenotransplantation.^18^ There are also fewer legal prohibitions against using pigs in research than there are against primates.^19^ Researchers are increasingly genetically modifying pigs set for transplant to make them less likely to be vectors of zoonotic diseases and to minimize rejection by transplant recipients.^20^

To avoid zoonoses, pigs destined for xenotransplantation are raised in “biosecure” environments before they're killed to harvest their organs. As numerous bioethicists have argued, there is a tension between maintaining biosecurity and promoting pig welfare.^21^ Pigs are intelligent, social creatures whose natural behaviors include rooting around in the dirt and foraging outdoors. They prefer particular companions and seem to attribute affective states to each other.^22^ They bond to their young, learn from each other, and like novelty in their play.^23^

But socializing spreads disease, and an outdoor environment is more difficult to carefully regulate and control. Often, pigs in biosecure facilities are delivered via cesarian section or quickly snatched from their mothers upon delivery, have their tails docked and their teeth clipped, and are kept in small groups in indoor “bubbles,”^24^ away from sunlight and grass and mud and places to engage in their natural behaviors. To be checked for organ health and disease status, they are subject to invasive procedures and extensive monitoring.^25^ For pigs, the cost of biosecurity is high.

## Animal‐centric Concerns: Permanent Large and Exploited Class

Xenotransplantation research poses the classical ethical conundrum of animal experimentation—exploitative and harmful use of animals in research aimed at human benefit. However, the goal of xenotransplantation research is the clinical adoption of xenotransplantation. And this makes the animal ethics concerns much more salient. The use of animals in this case is not just a step along the way to developing innovative medical treatments. Harmful use of animals is the end state. If xenotransplantation becomes an approved technology, large numbers of pigs would be exploited for the routine practice of clinical xenotransplantation. Accordingly, we predominantly focus our ethical analysis of xenotransplantation on the likely negative consequences of clinical application.

We now turn to the claim that xenotransplantation is harmful to pigs. Though it's controversial whether a painless death is a harm for animals, many philosophers claim it is, commonly arguing that death deprives animals of all the goods they could otherwise experience.^26^ Killing animals for food is often defended by appealing to the fact that those farmed animals would not exist at all if they weren't going to be consumed. In parallel, xenotransplantation might be defended by appealing to the fact that the bioengineered pigs would not exist at all if they weren't going to be killed for their organs. Versions of this problem have generated massive literatures in population ethics, animal ethics, and procreative ethics. We will not do them justice here but will give a case that addresses the objection well enough for our purposes. Taking inspiration from Gregory Kavka,^27^ imagine a woman with a high chance of heart failure later in life. She decides to have a child in order to one day kill them and get their heart transplanted into her. She plans, until that point, to give them a good life. This is obviously abhorrent. And while, of course, there are differences between persons and animals that matter for how humans treat them, we should be suspicious of the claim that bringing a creature into existence in order to kill it means the killing does not harm them or suffices to make the killing permissible.

Moreover, intuitively, almost all of us recognize that there is *something* wrong with killing sentient creatures. Though we might be willing to kill for good reason (and granted, giving a human many more years of life seems like the right sort of reason), that we think a compelling reason is required shows that we think there is some reason against killing that must be outweighed. Those who work in slaughterhouses suffer severely psychologically from committing so much violence.^28^ We wouldn't expect them to feel distress at dismantling robots. Plausibly, this difference is because they sense that animals matter morally and that they do something wrong in killing them.

Even if death itself isn't a harm for pigs, the practices required for adequately safe xenotransplantation are undeniably bad for them. As discussed above, guarding against zoonoses is in tension with adequately free, social, and stimulating lives for pigs. It is clearly better for a piglet to snuggle with its mother and run free in the grass than to be pulled from its mother upon delivery and kept in a cage under fluorescent lights. If the practices are bad enough to make the pigs’ lives not worth living, bringing them into existence is most obviously a wrong. If factory farming is any lesson, there is ample reason to worry that conditions will become worse for pigs as production scales up. When people make a profit by selling animals’ bodies, there is an incentive to improve profit margins by minimizing the space and care allotted per animal.

To create a huge, long‐lasting class of sentient, intelligent creatures who exist only for humans to take their organs is undeniably exploitative. As Martha Nussbaum says, in arguing that we ought to continue some medical experiments on animals although it violates their rights, “We should admit, then, that there will be an ineliminable residue of tragedy in the relationships between humans and animals.”^29^ But there is some level of tragedy that we cannot permissibly enact. In light of the continuing and large‐scale exploitation of pigs that would be required for clinical application, we believe that xenotransplantation is impermissibly tragic.

***Xenotransplantation with “disenhancement”?*** In response to concerns about xenotransplantation's effect on pig welfare, scientists could consider engineering pigs whose well‐being is less fragile. Philosophers like Adam Shriver have long advocated for disenhancement of farmed animals.^30^ Bioethicists have taken up this mantle for xenotransplantation, suggesting that pigs be bioengineered to be devoid of consciousness,^31^ or to have less capacity to suffer.^32^ However eerie these prospects are, they seem certainly better than xenotransplantation using suffering pigs. But although xenotransplantation is here, disenhancement remains beyond technological grasp. Given the psychological and genetic complexity of pain, anxiety, loneliness, and boredom, as well as the important functions they serve in keeping animals healthy, disenhancement could be decades away. In the meantime, xenotransplantation would result in tremendous suffering.

***Objection from lesser concern.*** When concerns about animal welfare are raised about xenotransplantation, many, including some who work in the burgeoning xenotransplantation industry, point to the use of animals in agriculture.^33^ Surely, raising pigs for life‐saving organs is more justified than raising them for sausages. This point is echoed in the literature on xenotransplantation.^34^ Not only is the purpose more compelling, since we can survive without meat but not without a heart, but xenotransplantation facilities plausibly will be less abysmal than factory farms. This is the newest iteration of an old argument. A century ago, John Dewey made a version of this point when defending animal experimentation by “scientific men.”^35^

There is something certainly right about these observations. Raising animals for food is typically worse than doing so for xenotransplantation. The conditions on factory farms are often horrific, and moral philosophers have long argued that using animals for food, especially when raising them in such conditions, is deeply wrong.^36^ But X's being better than related and very wrong Y does not make X permissible. To argue for X solely on this basis is to commit the fallacy of whataboutism.^37^ As we have argued, xenotransplantation is harmful and plausibly unjust to animals. That animal agriculture is worse does not mean that the concerns we have raised aren't sufficient reasons to avoid xenotransplantation as well.

Finally, we'll note that there can be strategic and logistical reasons to stop a practice X even as a similar but morally worse practice Y continues. It is often easier to avoid implementing a new harmful practice than it is to stop an existing one. The new practice doesn't have all the inertia behind it that the old one has or have so many who have organized their lives around it. The parties in control of practices X and Y might be differently amenable to reasons to halt the practices. Those in control of practice Y might be less invested in their practice or less powerful. It need not be unprincipled to try to stop X while knowing Y is worse. And it is certainly not unprincipled to say that we ought to halt xenotransplantation research *and* move away from animal agriculture.

For those skeptical of the animal‐directed reasons to halt xenotransplantation research and direct funds elsewhere, we turn now to human‐directed concerns.

## Humancentric Concerns: Justice in Research

The first person to receive a porcine heart, David Bennett, was deemed ineligible for a human heart by four transplant centers because of a history of “poor adherence to treatment.”^38^ As bioethicists have argued, there is something morally troubling about his selection for a xenotransplant. Not only are there concerns about the accuracy of predicting future noncompliance from past noncompliance, but that someone has trouble complying with medical guidance surely makes them an especially risky candidate for xenotransplantation.^39^ Because xenotransplant patients require increased immunosuppression and must be monitored for zoonotic disease, patient compliance is particularly important for both their safety and that of their families, communities, and the population at large.

So, who is often excluded from allotransplant lists, such that they might be willing, out of desperation, to undergo experimental xenotransplant? It will come as little surprise to anyone familiar with other inequities in the American health care system that there are disparities in transplant list exclusion. As Christine Park et al. found, “Racial and ethnic minorities, women, and patients in lower socioeconomic status groups were less likely to be referred, evaluated, and added to the waiting list for organ transplant.”^40^ The authors also found associations between “education, insurance status, geographic isolation from transplant center, and family support” and disparities in access.

People are ostensibly excluded from transplant lists because of medical criteria that affect how likely they are to survive transplant (or assumed proxies for these criteria like age or weight) or because of yet more nebulous determinations about how likely they are to comply with posttransplant treatment regimens. There are substantial problems with these criteria, both with how they are implemented and at a deeper philosophical level. Take the case of Dee Griffin. She “was told by a Maryland center that she was not a transplant candidate five years ago because she had a ‘pattern of non‐compliance,’ among other issues, according to medical records. She found that absurd, pointing out that she was at that time undergoing home dialysis, a process that requires patients to diligently adhere to the treatment.”^41^ Griffin had surely proven she was capable of complying with treatment. And indeed, being on dialysis at all, whether at home or by visiting a clinic multiple times a week, provides substantial evidence that a patient can, with the right support, “comply.”

There are, of course, patients who struggle to stick to a treatment plan. But what is really helpful for getting such patients to adhere to strict regimens is support. Xenotransplantation trials have money and time to invest in supporting patients. Research teams can make home health care visits and provide transport and dietary support. With the money spent on a few xenotransplantation trials, how many patients could instead be better supported through allotransplant or through managing conditions earlier so a transplant is not required? Of course, funding for trials is not easily redirected toward care. Better supporting struggling patients at a large scale requires systemic changes to our health care system. But this is a reminder not to treat adherence to treatment as immutable. Our society can invest in supporting adherence just as we invest in new technologies.

It should not be surprising that those who agree to xenotransplantation have been excluded from allotransplant. Absent a fanatical commitment to scientific progress or a saintlike desire to leave human organs for other recipients, no one would choose to experimentally receive a pig heart if they had clinical access to a human heart. Xenotransplant, unlike allotransplant, doesn't have a history of success. All four individuals who received pig organs in 2024 died within two months. And while nearly 95 percent of nonhuman primate allotransplant recipients are alive a year after transplant, this number falls to only 36 percent when the organ is from a pig, even with the best immunosuppression and genetic edits available.^42^ And though all transplant comes with risk of donor‐driven infection in the months following surgery, these risks are heightened with xenotransplantation. Less is known about how to monitor, identify, and treat zoonotic diseases, and so researchers have recommended “increased intensity of immunosuppression,” more extensive and invasive monitoring of xenotransplantation patients, and caution for their close contacts.^43^ The second person to receive a pig kidney, Lisa Pasano, spoke movingly of wanting to see her grandchildren grow up. “Any time on this Earth is better than none,” she observed. “So if I get two years, that's two years that I didn't have before.”^44^ We're not interested in condemning a transplant team trying to give a patient who is out of clinical treatment options more time to get to know their grandchildren. Public health measures come too late for many patients, and lab‐grown organs remain beyond reach. An individual researcher doesn't have the power to change waiting‐list standards, nor can they manifest a suitable living donor out of thin air. But patients must know what they are agreeing to, including being informed that, after undergoing the invasive surgery, previous patients have lived only a couple months.

But while we think there is reasonable disagreement about the appropriateness of offering xenotransplantation to those without clinical treatment options, we have significant reservations about the offering of this option, with its current track record, to those who might otherwise receive a human organ. This gives us great concern about one group of participants that the biotechnology corporation United Therapeutic says that they will include in their FDA‐approved xenotransplantation trial. While one group has been deemed ineligible for allotransplant, the other group consists of “patients who have been on the kidney transplant waitlist but are more likely to die or go untransplanted than receive a deceased donor kidney transplant within five years.”^45^ It seems insurmountably difficult to predict that with sufficient accuracy. Will participating in the trial make participants ineligible to receive a human organ, should it turn out that one does become available for them? That seems too steep a price for patients who have a reasonable chance at getting a successful allotransplant.

## Humancentric Concerns: Justice in Clinical Adoption

We now turn to issues of justice raised by the clinical adoption of xenotransplantation. Given its track record, and the anatomical and immunological dissimilarity of pig and human organs, there is ample reason to worry that xenotransplantation will have worse clinical outcomes than allotransplantation. Claims about how xenotransplant and allotransplant outcomes might eventually compare are highly speculative at this stage, and we are not best equipped to evaluate them. Instead, we will explore some ethical pitfalls of each possibility, some of which can be guarded against with enough foresight and political will. Further muddying the picture, however, is that, even if xenotransplantation could get as good as allotransplantation, how it compares could remain unknown for a long time.

If xenotransplantation's prospects are so poor that it never much improves beyond its current state, the opportunity costs of investing in xenotransplantation are especially concerning. That this is a possibility ought to weigh in a moral calculus and gives some reason to prefer more reliable preventive methods.

The ethical challenges individuals and society face should xenotransplantation improve yet remain less clinically successful than allotransplant are especially complicated. Without very careful regulation, there is substantial potential for such xenotransplantation to widen health disparities. There are laws against buying human organs, but no laws against buying pig organs. There is even some inconsistency with enacting such a law, given that we are permitted to buy pig parts to eat. Those with sufficient wealth could bypass allotransplant waiting lists (or insurance standards for xenotransplantation eligibility) by purchasing a bioengineered pig organ. If xenotransplantation is clinically similar to or even better than allotransplantation, there are clear incentives to do this. But even if pig organs remain less long‐lasting or otherwise desirable, this could still create a bridge to allotransplant available for only a few. A wealthy individual might purchase a series of pig organs, for instance, while insurance might limit how many they will cover.

There are more troubling ways xenotransplantation could widen health disparities. It could widen disparities not solely by improving outcomes for the wealthy, but also by worsening outcomes for the disadvantaged. Imagine that a very well‐off person offers to buy a pig heart for anyone higher than them on the allotransplant waiting list in their local area. This would be a hard offer for very sick patients to refuse, as they might reasonably worry that a human organ won't come for them in time. While this would clearly benefit those who would have died on the waiting list, such offers could make some absolutely worse off. For those who otherwise would have received a human organ had they waited longer, receiving a pig organ, if it requires more immunosuppression, doesn't last as long, or makes them so sick they are ineligible for allotransplantation, would make them worse off than they otherwise would have been. And these people will be the ones whom would‐be list‐hoppers have the greatest incentive to target with offers of paid‐for pig organs, given that getting them off the waiting list would do the most to increase the hoppers’ own human‐organ chances. While such scenarios are clearly not inevitable for clinical xenotransplantation, we call them to attention to illustrate unobvious potential abuses that must be guarded against. It is imperative to guard against such possibilities before any clinical adoption of xenotransplantation. Establishing regulations only after abuses have been committed leaves those who need transplants vulnerable to egregious exploitation.

Finally, we'll turn to a few considerations of justice that remain relevant even if xenotransplantation could eventually be as clinically successful as allotransplantation. First, the availability of more organs will not save people who are uninsured or unable to see a doctor due to a lack of reliable transportation—the very same people most at risk for developing organ failure. As Keisha Ray points out, xenotransplantation doesn't help Black men access primary care physicians, even though this is a prime reason that they have such a high rate of cardiovascular disease and untreated hypertension.^46^

This critique gets at two fundamental ways xenotransplantation is a less just solution to the organ crisis than alternatives like investment in prevention. Xenotransplantation is an intervention too far down the line to best change health outcomes for those with the least health care access. As Christopher Bobier et al. insightfully argue, if the underlying causes of organ failure are not addressed, a transplant alone will not necessarily improve one's health, especially over the longer term.^47^ And organ failure and transplant, even absent time on a waiting list, are a harrowing ordeal. It's preferable, at the individual level, to avoid needing an organ in the first place.

Ray's critique also calls to mind our earlier point about the opportunity costs of xenotransplantation. Justice requires some attention to efficiency and trade‐offs. The huge economic investment being poured into xenotransplantation has the potential to more justly and efficiently address the organ crisis if invested elsewhere.

## Alternatives to Xenotransplantation

There are some promising, familiar alternative interventions. Philosophers, bioethicists, and public health officials have long been making the case for allowing payment for organs and preventing kidney failure through public health interventions. One might, in light of the duration of most of these proposals, wonder why it's worthwhile to look at them again. After all, xenotransplantation doesn't seem to alter the ethics of moving to opt‐in organ donation, for instance. And whatever the ethics of these alternatives are, after all these years, they have not come to fruition.

Furthermore, as bioethicists have pointed out, there is a lot of money to be made selling bioengineered pig organs, and there is good reason to think this will create perverse incentives in the transplant system.^48^ As they argue, biotechnology companies have tremendous incentive to push for xenotransplantation, even if it delivers subpar results. Given all this, we recognize that the reader might think we are being unreasonably optimistic in trying to direct resources away from xenotransplantation.

For bioethicists, this situation is familiar. Bioethicists give the best arguments they can for the policies they believe to be the most ethically sound, and then they hope the public, advisory boards, and elected officials give them uptake. And nevertheless, we, the authors, think there is some reason to be hopeful. The news coverage of xenotransplantation has brought the organ crisis back into the public eye. Journalists have written moving accounts of individuals struggling and dying on organ waiting lists. Additionally, there is growing dissatisfaction with the band‐aid approach to health in the United States. All this gives us some hope that our society might muster the collective political will to really invest in addressing the organ crisis and to take seriously options like prevention.

***Public health interventions.*** Exciting new technological interventions like xenotransplantation can pull attention away from more effective but less exciting strategies like prevention.^49^ Most organ failure is preventable, caused by diseases like hypertension and type II diabetes that can be prevented with lifestyle interventions and inexpensive medical treatments. There is good evidence about which public health interventions would dramatically decrease the number of organ transplants needed. The health care system can screen those at high risk earlier to prevent disease progression, invest in maternal health, and help people access the tools to manage diabetes and hypertension and to quit smoking.^50^ Getting enough exercise and keeping hydrated are key to keeping one's kidneys working.^51^ As a society, we could make low‐sodium food more accessible, incentivize making cities more walkable, and subsidize gym memberships. We could let people take time out of their workdays to exercise, build more public water stations, and incentivize companies to lower the sugar content of their beverages. Additionally, glucagon‐like peptide‐1 inhibitors show great promise for helping patients manage chronic conditions such as diabetes and high blood pressure and thus reduce the risk of organ failure.^52^ Although expensive, these new interventions offer substantial benefits in health and quality of life.

Preventive interventions are important not only because they'll minimize both transplant surgeries and deaths but also because they help people to live more flourishing and healthier lives and prevent people from suffering the more immediate effects of failing organs. Kidney disease decreases quality of life for both sufferers and their caregivers; it can cause mobility and sleep problems, pain, itching, and other discomfort and can give rise to the need for burdensome and expensive dialysis.^53^

Of course, public health interventions will not eliminate all need for organ transplantation. Even with excellent public health interventions, some people will still struggle with alcohol‐use disorder or diabetes and will subsequently develop organ failure. But to address the organ crisis, public health interventions need not prevent all organ failure. Given current transplant numbers, getting to less than 46,000 failures in the United States a year would completely close the gap. As most organ failure is caused by preventable conditions, with substantial investment in public health interventions, this goal is not as unrealistic as it may initially seem.

There are two levers for addressing the organ crisis: lowering the need for organs and increasing organ availability. At the same time as public health interventions are working to lower the need, steps can be taken to increase the supply. We'll now turn to how this might be done without implementing xenotransplantation. Some methods of increasing the supply remain far off, yet others could be implemented more quickly.

***Improving organ donation.*** Though the vast majority of people support organ donation, only about half of Americans are registered organ donors.^54^ Nearly 50,000 transplant surgeries occurred in 2023.^55^ If everyone registered, this would approximately double potential donors and certainly go a substantial way to addressing the U.S. organ crisis.

Although there is disagreement about how effective moving to an opt‐out system alone is for increasing donation rates,^56^ modeling indicates that it could allow for over 17,000 more transplants in the U.S. each year.^57^ Mandatory posthumous donation, it follows, would result in even more. There are, of course, moral concerns with mandating donation. Though it's controversial whether decisions about our bodies after death fall properly under the scope of our bodily autonomy, it's plausible that our special relationship with our body entails that people ought to respect our wishes for it even after our death.^58^ Less contentiously, mandating donation risks inflicting psychological harm. Some individuals fear, in part due to a history of discrimination at the hands of the medical establishment, that being an organ donor means doctors won't try as hard to save their lives. Others have religious concerns about organ donation. Some Catholic bioethicists, for instance, have expressed concern that the brain‐death criteria used for organ donation conflicts with requirements of their faith.^59^

Given that the most needed organ is the kidney and kidneys can come from living donors, pursuing strategies to increase live donation could substantially ameliorate the organ shortage. Some argue that people ought to be allowed to sell one of their two kidneys.^60^ Extreme care would be required to set up a regulated payment system that would not facilitate organ trafficking or exploit those experiencing financial hardship. This might be done by issuing a tax credit or by paying people in yearly installments.^61^

While allotransplant involves costs to some people, it's not the case that any harm to humans is worse than *any* harm to *any number* of pigs. A person is harmed less, should their organs be used upon their death, than a pig is if it's forced to live a life indoors and is prematurely killed. Moreover, many of the costs of allotransplant are endured willingly. Many living donors report that donating boosted their self‐esteem, made their life more meaningful, or brought them closer to the loved one to whom they gave an organ.^62^ Many families take great comfort in knowing that their deceased loved ones’ organs allow someone else to live. Pigs, of course, do not feel as if their lives are made more meaningful by organ donation, nor do they go willingly to their death.

In addition to improving donation numbers, the United Network for Organ Sharing and health care systems can improve organ transport and management. There are reports of kidneys getting lost in airline cargo holds or stuck in closed cargo offices^63^ and of organs arriving at transplant centers frozen or squished.^64^ Well‐meaning policy changes, like prioritizing sicker patients over those who live close to transplant centers, can increase the number of kidneys that go to waste.^65^ In the United States, thousands of donated kidneys are thrown out because they're biopsied and determined to be “low‐quality” organs. These determinations are not systematic, and there's evidence that many discarded kidneys could be successfully transplanted with good long‐term survival rates.^66^ Surely, more systematic standards should be developed. And if xenotransplantation is to be adopted clinically, surely pig organs must not be held to more lax standards while comparatively longer‐lasting human organs get thrown out.

***Lab‐made organs.*** As effective as public health interventions and improved organ donation might be, such “low‐tech” solutions alone aren't the answer. Even with excellent public health interventions, many people will experience organ failure. Any time on a transplant waiting list is physically and emotionally difficult. Organs donated from others come with prior wear and some immunological mismatch. Lab‐made organs, either grown or 3D printed, could be without those weaknesses. Many proponents of xenotransplantation, in fact, see lab‐made organs as the ultimate goal.^67^

Lab‐grown organs, if grown with a patient's own immunological markers, wouldn't require burdensome immunosuppression. Scientists have been able to grow “relatively simple tissues such as skin, bladders, vessels, urethras, and upper airways” for fifteen years^68^ and “organoids of brain, intestine, lung, liver and numerous other organs are routinely generated now and used for purposes ranging from basic research to drug screening.”^69^ Lab‐grown blood cells have been successfully transfused.^70^

The process of 3D bioprinting could produce organs much faster than biological organisms grow them. Some body parts are already being successfully bioprinted. A cancer survivor has received the first 3D‐printed windpipe, which required no immunosuppression, and, as of this six months later, was doing well.^71^ But creating full, functioning organs remains a challenge. Though some researchers are optimistic that there will be 3D‐printed organs for transplant within a decade, many think that within twenty or thirty years is more likely.^72^ Xenotransplantation must not be allowed to crowd out research into these more ethical technologies. Our society has a long history of complacency about the use of animals in food and medicine, and we must be vigilant against repeating this pattern.

## More Justifiable Paths Forward

Plunging hundreds of millions of dollars into xenotransplantation, especially given the substantial chance it won't compare well to allotransplantation and given the animal welfare concerns even if it does, seems unjustifiable in light of our alternatives. The clinical adoption of xenotransplantation would be long‐lastingly and inevitably exploitative. The use of animals is not a temporary steppingstone, as it is for the most justified use of animals in medical research, but fundamental to the intervention. Should xenotransplantation become safe and effective, the number of animals raised in conditions not conducive to their welfare and killed for their organs will skyrocket.

And not only will the adoption of xenotransplantation mean lasting exploitation for animals; there is also substantial potential for injustice in how these organs are distributed among humans. Xenotransplantation's expansion could both widen gaps in health outcomes and make some individuals—disproportionally disadvantaged ones—absolutely worse off. While injustice of this sort is not inevitable for xenotransplantation, avoiding it will require thoughtful, creative planning. Xenotransplantation's track record, rapidly accelerating pace, and potential to create perverse incentives in transplant systems don't inspire optimism.

With the first FDA‐approved xenotransplantation trials beginning this year, we stand at a scientific and moral precipice. In setting our research priorities and deciding if we will let biotech companies scale up their production of gene‐edited pigs, we must keep the injustices, inefficiencies, and harms of xenotransplantation in mind. We mustn't get swept up in the technological novelty of this ethically troubling route of addressing the organ crisis. We have more efficient and just tools in hand already and ethically superior technological innovations on the horizon.

## Acknowledgments

This research was supported, in part, by the Intramural Research Program of the NIH. We are grateful to Christine Grady, David Wasserman, and the journal's anonymous reviewers for their very helpful feedback on the manuscript.

## Disclaimer

The views, information or content, and conclusions presented do not necessarily represent the official position or policy of, nor should any official endorsement be inferred on the part of, the Clinical Center, the National Institutes of Health, or the Department of Health and Human Services.
